# Comparison between Fasting and Non-Fasting Cut-Off Values of Triglyceride in Diagnosing High Triglyceride in Chinese Hypertensive Outpatients

**DOI:** 10.3390/jcm12072539

**Published:** 2023-03-28

**Authors:** Yingying Xie, Peiliu Qu, Liling Guo, Jin Xu, Liyuan Zhu, Yangrong Tan, Tie Wen, Ling Liu

**Affiliations:** 1 Department of Cardiovascular Medicine, The Second Xiangya Hospital, Central South University, Changsha 410011, China; 2 Research Institute of Blood Lipid and Atherosclerosis, Central South University, Changsha 410011, China; 3 Modern Cardiovascular Disease Clinical Technology Research Center of Hunan Province, Changsha 410011, China; 4 Cardiovascular Disease Research Center of Hunan Province, Changsha 410011, China; 5 Department of Emergency Medicine, The Second Xiangya Hospital, Central South University, Changsha 410011, China; 6 Emergency Medicine and Difficult Diseases Institute, The Second Xiangya Hospital, Central South University, Changsha 410011, China

**Keywords:** hypertension, non-fasting, triglyceride, remnant cholesterol, blood lipid test

## Abstract

**Background:** Hypertension (HBP) often occurs together with hypertriglyceridemia which indicates elevated triglyceride (TG) and remnant cholesterol (RC) levels. Non-fasting (i.e., postprandial) blood lipid test after a daily meal has been recommended by the European Atherosclerosis Society (EAS). However, little is known about the difference between fasting and non-fasting cut-off values in assessing high TG (HTG) and high RC (HRC) in HBP outpatients. **Methods:** 225 Chinese outpatients with HBP, including 119 fasting patients (i.e., fasting group) and 106 non-fasting patients (i.e., non-fasting group) were enrolled in this study. Non-fasting levels of blood lipids at 2 h after a daily breakfast were also tested in 33 patients among the fasting group. Venous blood samples were collected. **Results:** The non-fasting group had significantly higher levels of TG and RC while lower levels of total cholesterol, low-density lipoprotein cholesterol, and non-high-density lipoprotein cholesterol than the fasting group (*p* < 0.05). According to the TG and RC cut-off values of the EAS, the percentages of HTG and HRC in the non-fasting group were 72.6% and 70.8%, respectively, whereas those in the fasting group were 57.1% and 52.9%, respectively. According to the cut-off value of marked HTG commonly used in the Chinese population in clinical practice, the percentage of marked HTG in the non-fasting group was 57.5%, whereas that in the fasting group was 34.5%. However, the percentages of HTG (57.6% vs. 51.5%) and HRC (51.5% vs. 51.5%) marked HTG (30.3% vs. 33.3%) in the fasting state and at 2 h after a daily breakfast in 33 outpatients did not reach statistical significance. **Conclusion:** Non-fasting blood lipid tests could find more individuals with HTG as well as those with marked HTG among Chinese outpatients with HBP. It indicates that non-fasting blood lipid tests are worth being recommended in patients with HBP.

## 1. Introduction

Once hypertension (HBP) coexists with dyslipidemia [1], all-cause mortality and cardiovascular mortality increase sharply [2]. The primary goal of cholesterol-lowering therapy is to decrease low-density lipoprotein cholesterol (LDL-C) to target levels, and the secondary goal is to reduce non-high-density lipoprotein cholesterol (non-HDL-C). Non-HDL-C is the total amount of cholesterol contained in lipoproteins other than high-density lipoprotein (HDL). In Chinese patients with HBP, the prevalence of hypertriglyceridemia was significantly higher than that of hypercholesterolemia [3]. The latest evidence suggested that the prevalence of dyslipidemia in Chinese adults was as high as 33.8%, of which the prevalence of hypertriglyceridemia was the highest, reaching 16.9% [4]. Hypertriglyceridemia represents an increase in the amount of triglyceride (TG)-rich lipoproteins (TRLs) and their hydrolytic products, remnant lipoproteins (RLPs). Compared with nascent TRLs, RLPs contain more cholesterol ester, have smaller diameters, and thus are regarded as atherogenic as LDL. As a portion of non-HDL-C, remnant lipoprotein cholesterol (i.e., remnant cholesterol, RC) level is positively associated with TG level [5]. According to the lipid-lowering guideline recommended by EAS, mild TG elevation (fasting TG ≥ 1.7 mmol/L) indicates that increased risk for cardiovascular disease and needs to be concerned. In fact, high TG (HTG) is usually diagnosed on the definition of marked TG elevation (TG ≥ 2.3 mmol/L) in clinical practice [5,6]. Therefore, it is necessary to routinely evaluate the levels of TG and RC in Chinese patients with HBP in clinical practice. 

Due to the long history of fasting blood lipid tests, it is generally accepted by Chinese doctors and patients [7]. Many studies had confirmed a close relationship between fasting LDL-C levels and coronary heart disease (CHD) [8,9]. However, a clinical study with a large population recently showed that non-fasting TG levels at 2 to 4 h had the strongest association with cardiovascular events rather than fasting TG levels [10]. In addition, elevated non-fasting TG, as well as RC levels, were associated with an increased risk of myocardial infarction, ischemic heart disease, and death in the general population cohort of Denmark [11]. Non-fasting blood lipid test after a daily meal has been recommended in routine clinical practice by the European Atherosclerosis Society (EAS). This recommendation could be helpful for the diagnosis of hypertriglyceridemia in Chinese outpatients who forget to keep the fasting state. 

In this study, we assessed the difference in the levels of TG or RC as well as the percentages of high TG (HTG), high RC (HRC), or marked HTG between the fasting and non-fasting outpatients with HBP. Furthermore, a questionnaire survey was also conducted to learn about the awareness and acceptance of non-fasting blood lipid tests in Chinese outpatients with HBP.

## 2. Patients and Methods

### 2.1. Study Subjects

Two hundred and twenty-five outpatients with primary HBP were enrolled in this study in the Outpatient Department of Cardiovascular Medicine Department, Second Xiangya Hospital, Central South University from July 2019 to March 2021. HBP was defined as a history of systolic blood pressure values ≥ 140 mm Hg and/or diastolic blood pressure values ≥ 90 mm Hg for at least 3 days [12,13]. 

According to the fasting state or not, there were 119 fasting patients (i.e., fasting group) and 106 non-fasting patients (i.e., non-fasting group) visiting the Outpatient Department. Among all patients, 97 patients accepted a questionnaire survey to learn about the awareness and acceptance of non-fasting blood lipid tests. Fasting patients were those with a fasting period of at least 8 h before the blood lipid test. Non-fasting patients were those with blood lipid tests within 4 h after the last meal. 

All of them were excluded from a history of secondary HBP, thyroid diseases, liver and kidney diseases, autoimmune diseases, mental diseases, cancer, or other severe medical diseases before getting involved. No one took oral hypolipidemic agents or other drugs that affected lipid metabolism. All patients continued to take antihypertensive drugs as usual and their blood pressure was monitored throughout the course. This study was approved by the Ethics Committee of the Second Xiangya Hospital of Central South University and informed consent was obtained from all participants. 

### 2.2. Specimen Collection

After at least 8 h of overnight fasting, venous blood samples were collected in 119 fasting outpatients with HBP. Moreover, venous blood samples were also collected at 2 h after a daily breakfast in 33 patients among 119 fasting outpatients according to their daily habits. 106 non-fasting patients accepted non-fasting blood lipid tests after a daily breakfast or lunch, and venous blood samples were collected within 4 h after a daily meal according to their daily habits [14]. 

### 2.3. Laboratory Assays

The serum was separated from the venous blood samples. Serum levels of TG and total cholesterol (TC) were measured by automated enzymatic assays, and those of LDL-C and high-density lipoprotein cholesterol (HDL-C) were determined by the direct method, on a HITACHI 7170A analyzer (Instrument Hitachi Ltd., Tokyo, Japan) by a laboratory technician who had no knowledge of this study. The detection kits were provided by Japan and Pure Pharmaceutical Industry Co. (Wako). Levels of RC and non-HDL-C were estimated by the following formulas, RC = TC − (HDL-C) − (LDL-C) and non-HDL-C = TC − (HDL-C), respectively [15].

### 2.4. Cut-Off Values to Determine HTG and HRC

According to the European joint consensus statement from the EAS [16], the fasting cut-off values to determine HTG and HRC are TG levels of 1.7 mmol/L and RC levels of 0.8 mmol/L, respectively. Their corresponding non-fasting cut-off values to determine HTG and HRC are TG level 2.0 mmol/L and RC level 0.9 mmol/L [5,17], respectively. Moreover, the fasting cut-off value to determine marked HTG commonly used in the Chinese population in clinical practice is TG levels 2.3 mmol/L. Its corresponding non-fasting cut-off value to determine marked HTG is TG level 2.66 mmol/L [18]. 

Recently, we reported that the non-fasting cut-off values to determine HTG and HRC were TG level 2.02 mmol/L [17] and RC level 0.87 mmol/L [5], and marked HTG was TG level 2.66 mmol/L [18], in Chinese subjects. 

In this study, the non-fasting cut-off values to determine HTG, HRC, and marked HTG recommended by EAS and/or reported in Chinese subjects were all selected to evaluate the percentages of HTG, HRC, and marked HTG in HBP outpatients.

### 2.5. Evaluation of Awareness and Acceptance of Non-Fasting Blood Lipid Tests in Chinese Outpatients

In order to learn about the awareness and acceptance of non-fasting blood lipid tests in outpatients with HBP, 97 patients were invited to answer two questions. Each question is a single-choice patient. Awareness evaluation referred to whether outpatients should keep a fasting or non-fasting state for blood lipid tests. There were four options, i.e., ‘fasting’, ‘non-fasting, ‘either is OK’, and ‘having no idea’. Acceptance evaluation referred to the investigation of the choice made by outpatients again after being told that both fasting and non-fasting lipid tests were available and recommended. There were four options, i.e., ‘fasting’, ‘non-fasting’, ‘either is OK’, and ‘depending on doctor’. 

### 2.6. Statistical Analysis

Quantitative variables of normal distribution were expressed as mean ± standard deviation (SD), and those of non-normal distribution were analyzed statistically after logarithmic conversion. Qualitative variables were expressed as numbers and percentages. The difference of the inter-group quantitative variables was analyzed by independent-samples *t*-test. Paired *t*-test was used for comparing quantitative variables between the two time points. Qualitative variables were compared using the Chi-square test for R × C. All statistical analyses were performed with SPSS version 26.0. All *p* values were 2-tailed, and *p* < 0.05 was considered statistically significant. 

## 3. Results

### 3.1. General Clinical Characteristics of Two Groups

There were 225 participants aged 18 to 70 recruited in this study population, and the fasting patients (i.e., the fasting group) and non-fasting patients (i.e., the non-fasting group) were found in 119 (52.9%) and 106 (47.1%) among the individuals, relatively. The general characteristics of the fasting group (*n* = 119) and the non-fasting group (*n* = 106) were presented in Table 1. There was no significant difference in age, gender, body mass index (BMI), waist, systolic or diastolic blood pressure, heart rate, percentage of central obesity, and proportion of patients with CHD and diabetes mellitus (DM). The number of current smokers in the non-fasting group was significantly lower than that in the fasting group (*p* < 0.05).

### 3.2. Comparison of Blood Lipid Levels between Two Groups

**Figure 1:**
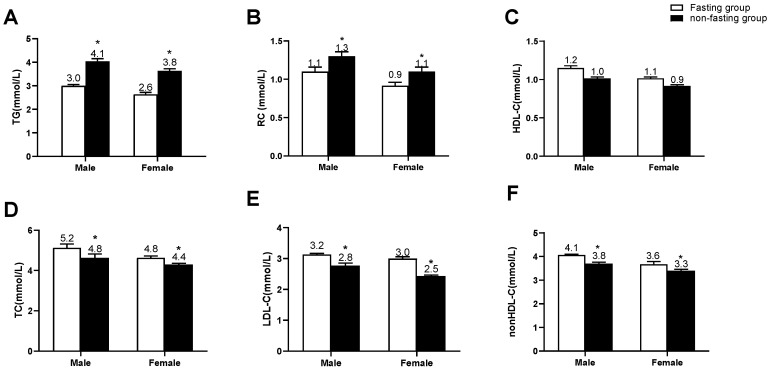
Comparisons of serum levels of blood lipids according to the gender between the two groups. (**A**–**F**) Serum levels of TG, RC, HDL-C, TC, LDL-C, and non-HDL-C in the fasting group (male, *n* = 80; female, *n* = 39) and non-fasting group (male, *n* = 71; female, *n* = 35), respectively. The bar represents standard error of the mean. * p < 0.05 when compared with the fasting group.

Among 106 non-fasting outpatients, 42 patients (39.6%) visited the doctors after breakfast whereas 64 patients (60.4%) visited the doctors after lunch. Furthermore, 62 patients (58.5%) were tested at 2 h, 18 patients (17.0%) were tested at 3 h, and 26 patients (24.5%) were tested at 4 h after a daily breakfast or lunch. Among 62 patients who were tested at 2 h, 28 patients (45.2%) were tested after breakfast whereas 34 patients (54.8%) were tested after lunch. Among 18 patients who were tested at 3 h, 7 patients (38.9%) were tested after breakfast whereas 11 patients (61.1%) were tested after lunch. Among 26 patients who were tested at 4 h, 12 patients (46.2%) were tested after breakfast whereas 14 patients (53.8%) were tested after lunch (Figure S1). Compared with the fasting group, the non-fasting group had significantly higher TG and RC levels (*p* < 0.05) whereas lower levels of TC, LDL-C, and non-HDL-C (*p* < 0.05). There was no significant difference in HDL-C levels between the two groups (Figure 1A–F).

### 3.3. Comparisons of Percentages of HTG, HRC, and Marked HTG between Two Groups

**Figure 2:**
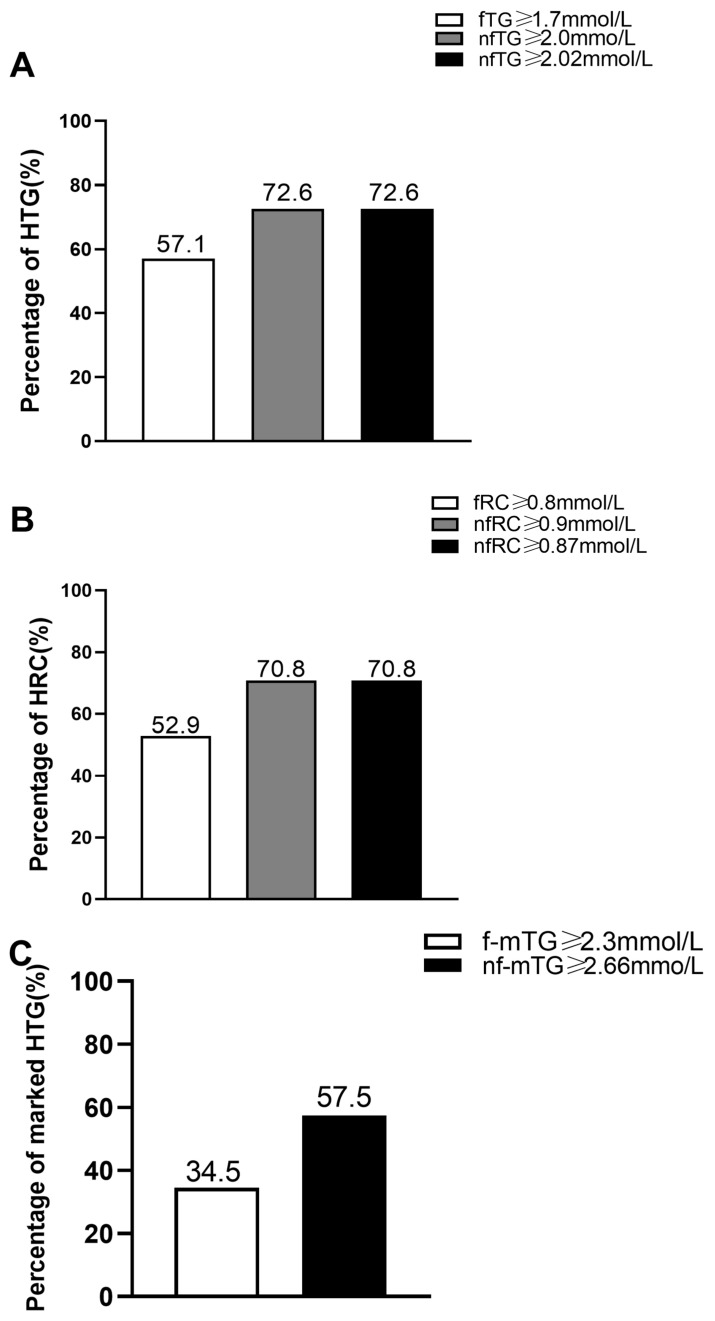
Comparisons of the percentages of HTG, HRC and marked HTG between fasting group (*n* = 119) and non-fasting group (*n* = 106). (**A**) Comparison of HTG according to the cut-off points of fasting TG (fTG) ≥ 1.7 mmol/L, non-fasting TG (nfTG) ≥ 2.0 mmol, and nfTG ≥ 2.02 mmol/L, respectively. (**B**) Comparison of HRC according to the cut-off points of fasting RC (fRC) ≥ 0.8 mmol/L, non-fasting RC (nfRC) ≥ 0.9 mmol/L, and nfRC ≥ 0.87 mmol/L, respectively. (**C**) Comparison of marked HTG according to the cut-off points of fasting marked HTG (f-mTG) ≥ 2.3 mmol/L, non-fasting marked TG (nf-mTG) ≥ 2.66 mmol/L.

According to the cut-off values of HTG and HRC recommended by the EAS [14], the percentages of HTG and HRC were 57.1% and 52.9%, respectively, whereas those in the fasting group were 72.6% and 70.8%, respectively. According to the fasting cut-off value commonly used in the management of marked HTG in the Chinese population, the percentage of marked HTG in the fasting group was 34.5%. According to the non-fasting cut-off values to determine HTG, HRC, and marked HTG reported in Chinese subjects [15,16,17], the percentages of HTG, HRC, and marked HTG in the non-fasting group were 72.6%, 70.8%, and 57.5%, respectively (Figure 2A–C).

### 3.4. Comparisons of Blood Lipid Levels and Percentages of HTG, HRC, and Marked HTG before and after a Daily Meal in 33 Outpatients

**Figure 3:**
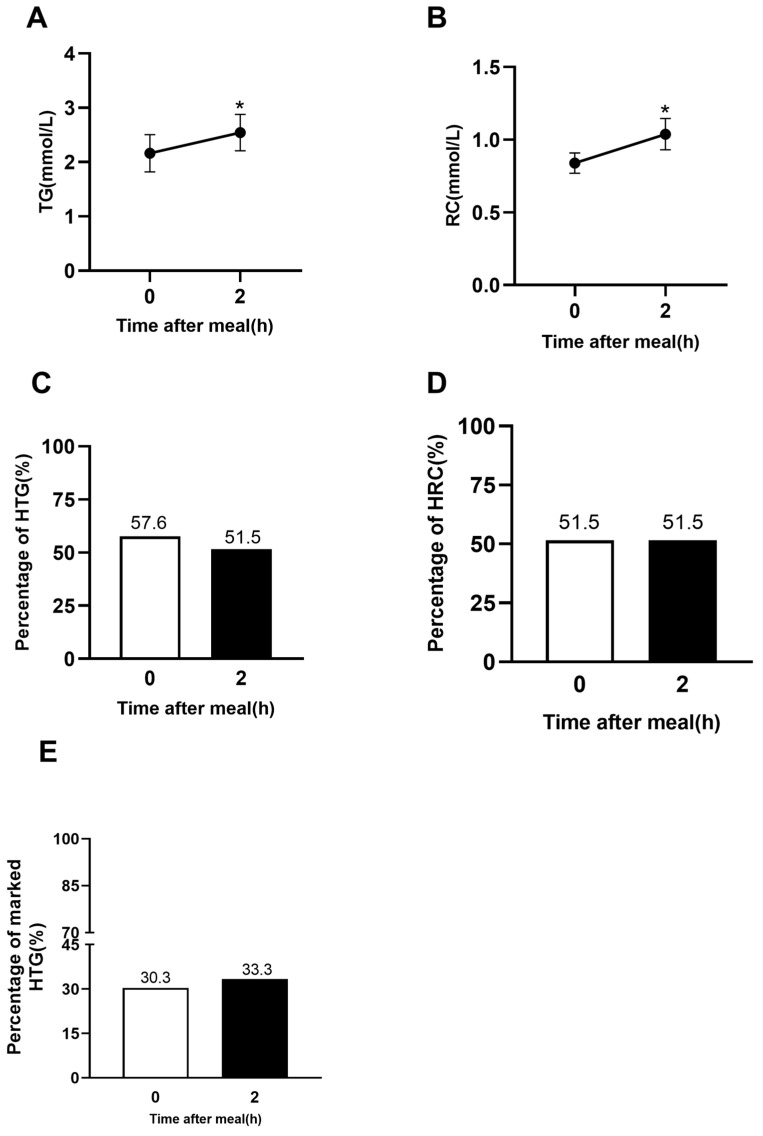
Changes in serum levels of blood lipids and comparison of percentages of HTG and HRC in the outpatients with HBP (*n* = 33). (**A**,**B**) Non-fasting changes in serum levels of TG and RC between 0 h and 2 h after a daily meal. The bar represents standard error of the mean. (**C**–**E**) Percentages of HTG, HRC, and marked HTG at 0 h and 2 h after a daily meal according to nfTG ≥ 2.0 mmol/L, nfRC ≥ 0.87 mmol/L, nf-mTG ≥ 2.66 mmol/L, respectively. * *p* < 0.05 when compared with the fasting level.

In 33 outpatients, non-fasting levels of TG and RC at 2 h after a daily meal were significantly higher than fasting levels of TG and RC (*p* < 0.05, Figure 3A,B). According to the cut-off values of HTG and HRC recommended by ESC [14], the percentages of HTG and HRC in the fasting state were 57.6% and 52.9%, respectively, and those in the non-fasting state were 51.5% and 51.5%, respectively. Additionally, the percentage of marked HTG in the fasting state was 30.3%, and that in the non-fasting state was 33.3%. There was no significant difference in the percentages of HTG, HRC, or marked HTG between the fasting state and non-fasting state at 2 h after a daily meal (Figure 3C,D). 

### 3.5. Awareness and Acceptance of Non-Fasting Lipid Test

**Figure 4:**
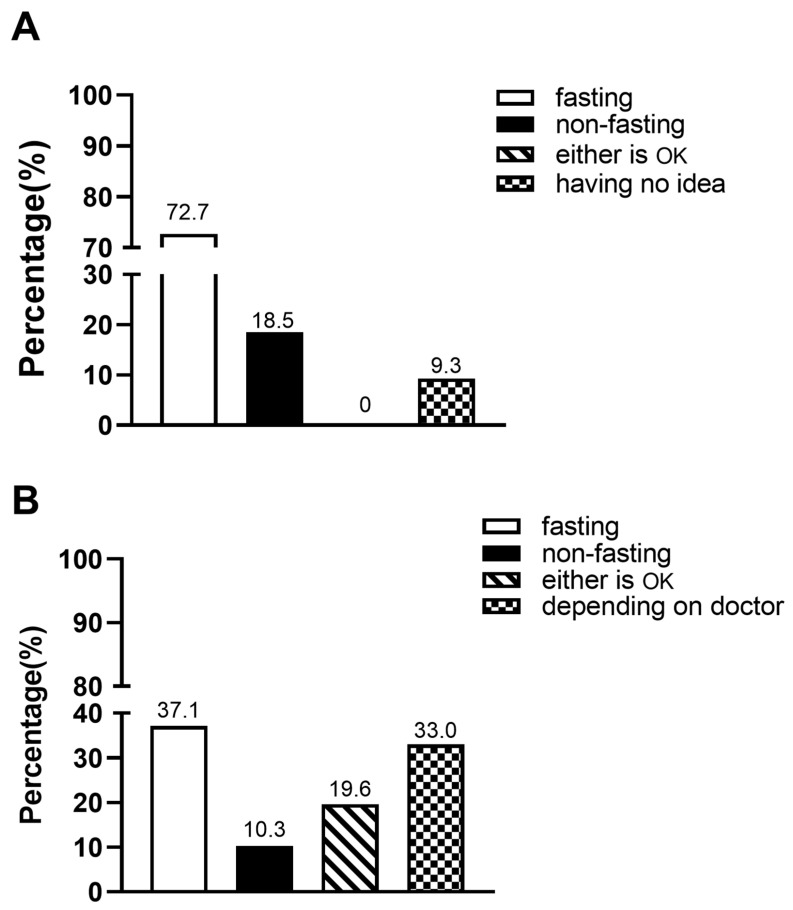
Awareness and acceptance of non-fasting blood lipid test. (**A**) Awareness of non-fasting blood lipid test in HBP outpatients. (**B**) Acceptance of non-fasting blood lipid test in HBP outpatients after patient education.

For the first question about awareness of non-fasting blood lipid test, 72.7% of outpatients chose the option of ‘fasting’, 18.5% chose the option of ‘non-fasting’, no one chose the option of ‘either is OK’, and 9.3% chose the option of ‘having no idea’ (Figure 4A).

For the second question about acceptance of non-fasting blood lipid test after immediate notification, 37.1% of outpatients chose the option of ‘fasting’, 10.3% chose the option of ‘non-fasting’, 19.6% chose the option of ‘either is OK’, and 33.0% chose the option of ‘depending on doctor’ (Figure 4B).

## 4. Discussion

In this study, prominently elevated levels of TG and RC were found in the non-fasting group when compared with those in the fasting one, which is similar to the finding of the studies with a large-scale general population in Denmark [5,10,18]. In those Danish studies, the comparison between fasting and non-fasting blood lipid levels was carried out in different subjects according to the time of visiting the clinics [5,18]. The significant elevation of non-fasting TG and RC levels after a daily meal was also observed in 33 fasting outpatients with HBP in this study. Moreover, the mean TG level of the fasting outpatients was more than 1.7 mmol/L and that of the non-fasting outpatients exceeded 2.0 mmol/L, indicating the augmented synthesis and/or reduced elimination of TRLs and their remnants after a daily meal in Chinese patients with HBP.

Conversely, the non-fasting group had significantly lower levels of TC, LDL-C, and non-HDL-C than the fasting group, which was similar to the finding of a Danish study carried out in different subjects according to the time of visiting the clinics [13]. We recently observed the non-fasting reduction in levels of TC, LDL-C, and non-HDL-C in inpatients who were overweight, had coronary heart disease, or both [5,18,19]. There were several possible explanations for the non-fasting reduction in those lipid parameters. First, the decrease in non-fasting levels of TC, LDL-C, and non-HDL-C was likely due to hemodilution caused by dietary fluid intake [20,21]. Second, increased TRLs and their remnants after a daily meal could activate cholesteryl ester transporter protein in the case of hypertriglyceridemia, which ultimately increased RC levels and decreased LDL-C through promoting the transfer of cholesteryl ester from LDL to TRLs and their remnants [22,23]. It is noteworthy that there was no significant difference in HDL-C levels between the two groups, which was consistent with the results of other researchers who confirmed that HDL-C level was not affected by food intake [13]. 

We recently reported that the non-fasting cut-off values to determine HTG or HRC in Chinese subjects were very close to those recommended by the EAS [14], which induced that the corresponding percentages of non-fasting HTG or HRC dependent on those values were also very similar. Compared with the fasting group, the non-fasting group exhibited higher percentages of HTG, HRC, and marked HTG. Although the difference in the percentages of HTG, HRC, or marked HTG between the non-fasting group and the fasting one could not be statistically analyzed, the higher percentage of HTG and marked HTG in the non-fasting group still suggested that non-fasting blood lipid test could increase the probability of detecting HTG in Chinese outpatients with HBP.

Interestingly, the difference in the percentages of fasting and non-fasting HTG was small and insignificant in 33 outpatients with HBP. In addition to the small sample size, another cause for this phenomenon could be the difference in the time of the blood lipid test. We previously found that levels of TG and RC began to rise at 2 h and peaked at 4 h after a daily meal in Chinese patients with coronary heart disease and HBP [14,15]. For some patients who are used to a high-fat and high-calorie diet, the peak TG level may be higher or later [24,25]. It indicated that serum TG level at 4 h after a daily meal can better reflect the maximum ability to synthesize and metabolize TG in a certain individual, whereas serum TG level at 2 h after a daily meal should be a relatively closer value to fasting level in Chinese subjects [13]. In the non-fasting group, about 44% of outpatients (*n* = 47) were tested at 2 h after a daily breakfast, and about 55% (*n* = 59) were tested later than 2 h after a daily breakfast or even after a daily lunch. It was consistent with the finding that the mean TG level in the non-fasting group was more than 3.0 mmol/L while that at 2 h in 33 outpatients was about 2.5 mmol/L. Moreover, it was reported that the average TG level after a daily lunch is higher than that at 2 h after a daily breakfast in Chinese subjects [26,27,28], which could be attributed to the additive effect of increasing TG by breakfast and lunch. Thus, TG level at 2 h after a daily meal may substitute its fasting value to evaluate HTG according to the non-fasting cut-off value for some special patients who are prone to hypoglycemia and physical weakness, such as diabetics, pregnant women, the elderly, and children. However, in order to improve the diagnostic positive rate of HTG or to detect the maximum response of TG increase after a daily meal, blood lipid tests at 4 h after a daily lunch could be more appropriate.

Chinese outpatients had little knowledge of the concept of non-fasting blood lipid tests. Among the surveyed outpatients, less than one in five ones chose non-fasting blood lipid tests. The vast majority of them chose fasting testing, which shows that this view has been widely accepted. After brief communication and notification on non-fasting blood lipid tests, nearly half of them turned to accept non-fasting lipid tests although more than one-third of them still insisted on fasting blood lipid tests. It was worth noting that about one-third of the surveyed outpatients left the decision to receive fasting or non-fasting blood lipid test to their doctors. That is to say, the application of non-fasting blood lipid tests in those subjects depends on their doctors. It indicates that the concepts of both doctors and patients must be updated through education in order to promote non-fasting blood lipid tests in clinical practice in China. 

There were several limitations in this study. First, the sample size was relatively small, especially compared with large-scale population studies abroad [9,10]. Second, given the crucial role of controlling LDL-C levels in the prevention of cardiovascular disease in HBP patients, non-fasting LDL-C levels also should be evaluated in HBP patients. Since the goal of LDL-C level in HBP patients depends on the corresponding cardiovascular risk determined by underlying diseases and risk factors, it will be more complex than the determination of non-fasting HTG, HRC, or marked HTG. In the future, prospective studies about non-fasting LDL-C levels to assess the risk of cardiovascular diseases in Chinese patients with HBP and guide clinical therapeutics are needed. 

## 5. Conclusions

Non-fasting blood lipid tests could find more individuals with HTG as well as those with marked HTG among Chinese outpatients with HBP. It indicates that non-fasting blood lipid tests are worth being recommended in patients with HBP. Considering the fact that the percentage of HTG at 2 h after a daily breakfast seemed to be close to that in the fasting state, the postprandial time-point at 2 h could be a more suitable substitute for the fasting one than that at 4 h when the fasting TG need to be assessed in the patients who visit their doctors in the postprandial state. 

## Figures and Tables

**Table 1:** Comparison of clinical characteristics between two groups.

	Fasting Group (*n* = 119)	Postprandial Group (*n* = 106)
Age (y)	45.5 ± 10.6	46.0 ± 9.6
Men, *n* (%)	80 (67.2)	71 (67.0)
BMI (kg/m^2^)	27.1 ± 3.7	26.4 ± 3.8
Waist (cm)	94.1 ± 10.4	94.9 ± 11.8
Current smoking, *n* (%)	32 (26.9)	15 (14.2) *
Central obesity, *n* (%)	88 (74.0)	75 (71)
CHD, *n* (%)	5 (4.2)	3 (2.8)
DM, *n* (%)	6 (5.0)	4 (3.8)
SBP (mm Hg)	157.2 ± 22.5	154.6 ± 19.5
DBP (mm Hg)	98.1 ± 15.8	102.5 ± 88.7
HR (bpm)	85.3 ± 14.7	88.7 ± 14.4

BMI, body mass index; CHD, coronary heart disease; DM, diabetes mellitus; SBP, systolic pressure; DBP, diastolic pressure; HR, heart rate; bpm, beats per minute; * *p* < 0.05 when compared with fasting group.

## Data Availability

The datasets analyzed during the current study are available from the corresponding author upon reasonable request.

## Supplementary Materials

The following supporting information can be downloaded at: https://www.mdpi.com/article/10.3390/jcm12072539/s1. Figure S1. Comparisons of the number of HBP outpatients with non-fasting lipid test at different time points after breakfast or lunch.
