# Supplementary material for: Comparison between Fasting and Non-Fasting Cut-Off Values of Triglyceride in Diagnosing High Triglyceride in Chinese Hypertensive Outpatients
Source: J Clin Med. 2023 Mar 28;12(7):2539. doi: 10.3390/jcm12072539 (PMC10095265; doi:10.3390/jcm12072539)
Supplement: Supplementary file 1 [file jcm-12-02539-s001.zip › jcm-2177221-supplementary.pdf]

Journal of
Clinical Medicine

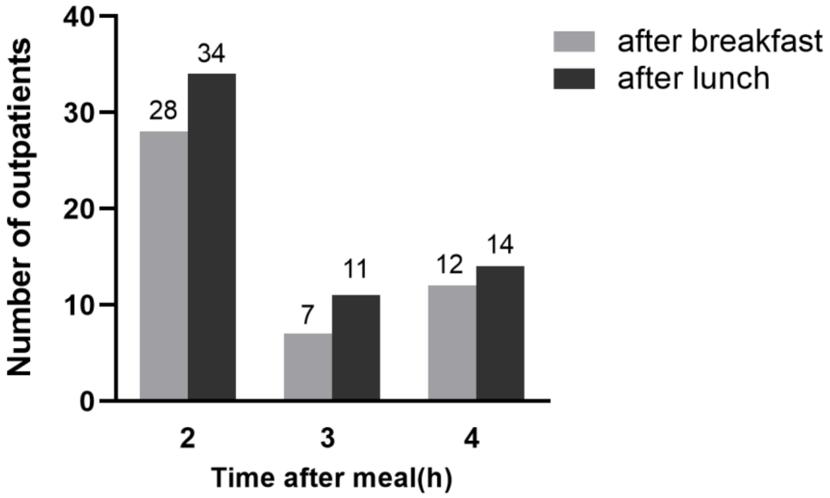

Figure S1. Comparisons of the number of HBP outpatients with non-fasting lipid test at different time points after breakfast or lunch.
